# PGC-1α and exercise intensity dependent adaptations in mouse skeletal muscle

**DOI:** 10.1371/journal.pone.0185993

**Published:** 2017-10-19

**Authors:** Nina Brandt, Maja Munk Dethlefsen, Jens Bangsbo, Henriette Pilegaard

**Affiliations:** 1 The August Krogh Club, Section for Cell Biology and Physiology, Department of Biology, University of Copenhagen, Universitetsparken 13, Copenhagen Ø, Denmark; 2 Section of Integrative Physiology, Department of Nutrition, Exercise and Sports, University of Copenhagen, Universitetsparken 13, Copenhagen Ø, Denmark; University of Birmingham, UNITED KINGDOM

## Abstract

The aim of the present study was to examine the role of PGC-1α in intensity dependent exercise and exercise training-induced metabolic adaptations in mouse skeletal muscle. Whole body PGC-1α knockout (KO) and littermate wildtype (WT) mice performed a single treadmill running bout at either low intensity (LI) for 40 min or moderate intensity (MI) for 20 min. Blood and quadriceps muscles were removed either immediately after exercise or at 3h or 6h into recovery from exercise and from resting controls. In addition PGC-1α KO and littermate WT mice were exercise trained at either low intensity (LIT) for 40 min or at moderate intensity (MIT) for 20 min 2 times pr. day for 5 weeks. In the first and the last week of the intervention period, mice performed a graded running endurance test. Quadriceps muscles were removed before and after the training period for analyses. The acute exercise bout elicited intensity dependent increases in LC3I and LC3II protein and intensity independent decrease in p62 protein in skeletal muscle late in recovery and increased LC3II with exercise training independent of exercise intensity and volume in WT mice. Furthermore, acute exercise and exercise training did not increase LC3I and LC3II protein in PGC-1α KO. In addition, exercise-induced mRNA responses of PGC-1α isoforms were intensity dependent. In conclusion, these findings indicate that exercise intensity affected autophagy markers differently in skeletal muscle and suggest that PGC-1α regulates both acute and exercise training-induced autophagy in skeletal muscle potentially in a PGC-1α isoform specific manner.

## Introduction

Endurance exercise training increases skeletal muscle oxidative capacity as evidenced by increases in the content of proteins in oxidative metabolism [1]. These metabolic adaptations seem to arise from cumulative effects of exercise-induced transient transcriptional responses. This is exemplified by studies reporting exercise-induced transient increases in transcription and/or mRNA content of metabolically related proteins in both rodent [2–5] and human [6–8] skeletal muscle.

The transcriptional coactivator Peroxisome proliferator-activated receptor-gamma coactivator 1 alpha (PGC-1α) has been suggested to be a key factor in mediating exercise training-induced adaptations in mitochondrial capacity [9–11]. Several studies using muscle specific PGC-1α knockout (MKO) and overexpression as well as whole body knockout (KO) mice [12–19] have underlined PGC-1α as a regulator of mitochondrial biogenesis. Thus, while 4 weeks of voluntary wheel running increased cytochrome c oxidase (COX) IV and Cytochrome c (Cyt c) protein in skeletal muscle in wildtype (WT) mice, PGC-1α MKO mice were able to increase COX IV to a less extent than WT mice but not Cyt c protein [19]. Furthermore, lifelong exercise training of WT mice prevented an age associated decline in Cyt C protein content, while mice lacking PGC-1α did not obtain this adaptation with exercise training [6,20]. However, when PGC-1α KO mice exercise trained by a combination of wheel running and treadmill running they were able to obtain a similar percentage increase in oxidative proteins in skeletal muscle as WT mice [14]. Furthermore, the findings that muscle specific overexpression of PGC-1α was associated with elevated Microtubule-associated protein 1A/1B-light chain 3 (LC3)I and II protein as well as reduced p62 protein in skeletal muscle provide evidence that PGC-1α also influences autophagy regulation (Lira et al 2013). In addition, studies showing PGC-1α dependent exercise-induced increase in the LC3 ratio (LC3II/LC3I) in skeletal muscle may suggest that exercise-induced autophagy is required for exercise training-induced metabolic adaptations and that these are mediated by PGC-1α [21–23]. Taken together this indicates that PGC-1α is a major player in the regulation of exercise training-induced adaptations, but that other factors also contribute. The previous studies [6,14] suggest that the role of PGC-1α in metabolic adaptations with exercise training may depend on exercise volume and/or intensity, but this remains to be determined.

PGC-1α is induced by acute exercise in rodent [14,24,25] and human [26] skeletal muscle. Furthermore, the exercise-induced increase in PGC-1α mRNA after exercise has been suggested to be affected by exercise intensity in humans [27–29]. Supporting that exercise intensity dependent PGC-1α regulation influences PGC-1α mediated exercise training adaptations. Multiple factors including Ca^2+^, reactive oxygen species (ROS) and adrenaline have been suggested as initiating factors leading to the regulation of PGC-1α transcription[10,30–38]. Furthermore the intracellular energy sensor AMP protein kinase (AMPK) is activated by phosphorylation during exercise [39] in an exercise intensity dependent manner [40] and has been suggested to regulate PGC-1α at the transcriptional level [4]. In addition, it has been suggested that AMPK regulates PGC-1α activity in skeletal muscle because in vitro experiments demonstrated that AMPK phosphorylates PGC-1α protein on two residues [41].

Transcription of PGC-1α in skeletal muscle is controlled by two promotor regions, the alternative promotor and the proximal promotor, together with alternative splicing, giving rise to different mRNAs, suggested to be translated into different PGC-1α isoforms [42]. The alternative promotor controls the transcription of PGC-1α b and PGC-1α c, which are characterized by two different versions of the novel exon 1b [42], whereas the proximal promotor controls the transcription of the originally discovered PGC-1α transcript [43], determined PGC-1α1/-a. In addition full length PGC-1α and N-terminal (NT) isoforms have been identified [44]. A previous study has shown both unique and similiar roles for full length PGC-1α and NT-isoforms in brown adipose tissue, suggesting different regulation of the PGC-1α isoforms [44]. In accordance a study reported that NT-PGC-1α is sufficient to induce thermogenesis in adipose tissue, when full length PGC-1α was lacking [45]. Furthermore, another study has reported that PGC-1α4/NT PGC-1α –b contributes in the regulation of skeletal muscle hypertrophy in response to resistance exercise training [46]. Moreover, it has been reported that a single bout of exercise increased the PGC-1α b and PGC-1α c mRNA levels, but not the PGC-1α a mRNA in mouse skeletal muscle [47]. Furthermore, exercise intensity has been suggested to affect which isoform transcripts are increased, as high intensity exercise has been shown to increase PGC-1α a mRNA, whereas low, medium and high intensity exercise increased PGC-1α b and PGC-1α c mRNA [48], and a study showed the same for the a, b and c NT-isoforms [49]. Therefore intensity-dependent PGC-1α isoform regulation may be involved in exercise-induced adaptations in skeletal muscle. However, a coupling between intensity dependent regulation of PGC-1α isoforms and exercise and exercise training adaptive responses remains to be explored.

Therefore the aim of the present study was to examine the role of PGC-1α in intensity dependent acute exercise and exercise training-induced adaptive responses in skeletal muscle.

## Methods

### Mice

Animal experiments were approved by the Danish Animal Experiment Inspectorate and complied with the European convention for the protection of vertebrate animals used for experiments and other scientific purposes (Council of Europe, no.123, Strasbourg, France, 1985).

Generation and characterization of the whole body PGC-1α KO and WT mice used in this study have previously been described [16]. Genotyping of the mice was performed by DNA extraction from either a tail or ear piece and fragment amplification by PCR using KO and WT specific primers as previously described [14]. Mice were kept on a 12:12 h light/dark cycle and had ad libitum access to water and standard rodent chow (Altromin no 1324; Brogården, Lynge, DK). All mice were single caged during the experimental period.

### Experimental protocols

#### Single treadmill exercise bout

Male and female KO and littermate WT mice, 12 weeks of age, were subjected to a single treadmill (Model Exer-4 Treadmill, Columbus Instrument; Columbus, OH, USA) running bout at either low intensity (LI) at 14 m/min and 10° incline for 40 min or moderate intensity (MI) at 18 m/min and 10° incline for 20 min. Mice were euthanized by cervical dislocation immediately after exercise (0h) or at 3h or 6h of recovery. Control mice, resting in the cage, were euthanized at the time points corresponding to 0h and 6h of recovery (n = 8). Trunk blood was collected following decapitation and quadriceps (Q) muscles were qwickly frozen in liquid nitrogen. Plasma was obtained by centrifugation at 2600 g for 15 min at 4°C. Samples were stored at -80°C until analyses were performed.

#### Exercise training

Male and female KO and littermate WT mice, 12 weeks of age, were exercise trained on a treadmill at 14 m/min and 10° incline (LIT) or 18m/min and 10° incline (MIT) 2 times pr. day for 5 weeks. In the first and the last week of the intervention period, the trained mice performed a graded running performance test at 10° incline and with the intensity beginning at 12 m/min increasing with 2 m/min every 5 min until exhaustion. Untrained mice served as controls (CON). Mice were euthanized by cervical dislocation 24h after the last exercise bout, (n = 10). Quadriceps muscles were quickly frozen in liquid nitrogen. Mice were MR scanned (EchoMRI^TM^, USA) two days before the euthanization. The moderat intensity protocol was chosen based on the running capacity of the PGC-1α KO mice, as they were not able to run at a higher intensity for 20 min.

### Analyses

#### Plasma analyses

Adrenaline levels were determined with an enzyme immunoassay (2-cat (A-N) Research ELISA^TM^, Labor Diagnostika Nord) following the manufacturer’s instructions.

Plasma lactate was measured fluorometrically as previously described [50].

#### Muscle analyses

Muscle samples was crushed in liquid nitrogen with a pair of pliers in order to secure homogeneity.

#### Muscle glycogen

Muscle glycogen content was determined on ~10 mg quadriceps muscle as glycosyl units after acid hydrolysis as previously described [50].

#### Citrate Synthase activity

Quadriceps muscle was homogenized in 0,3 M phosphate buffer (pH 7.7) containing 0.05% bovine serum albumin for 2 minutes at 30 oscillations per second in a TissueLyser (TissueLyser II, Qiagen, Valencia, CA, USA). Maximal Citrate Synthase (CS) activity was determined according to the manufacturer`s protocol (Sigma-Aldrich, St Louis, MO USA), with absorbance kinetically measured at 405 nm (Multiscan; Thermo Scientific) at baseline and after addition of oxaloacetate.

#### Muscle proteins

Muscle lysate was produced from ~20 mg quadriceps muscle by homogenization in ice-cold buffer (10% glycerol, 20 mM Na-pyrophosphate, 150 nM NaCl, 50mM HEPES, 1% NP-40, 20mM β-glycerophosphate, 10 mM NaF, 1 mM EDTA, 1 mM EGTA, 20μg/ml Aprotinin, 10μg/ml Leupeptin, 2mM Na3VO4, 3 mM Benzamidine, pH 7,5) for 2 minutes at 30 oscillations per second in a TissueLyser (TissueLyser II, Qiagen, Valencia, CA, USA). The samples were set to rotate end over end for 1h at 4°C followed by centrifugation at 17,500 g for 20 min at 4°C. The lysates were collected as the supernatant. The protein content in the lysates was determined by the bicinchoninic acid method (Pierce Chem, Comp., IL) and lysates were prepared with sample buffer containing Sodium Dodecyl Sulfate (SDS) and boiled for 3 min at 96°C. Phosphorylation levels and protein content were measured by SDS-PAGE and western blotting using self-casted gels. PVDF membranes were blocked in 3% fish gel, and protein and phosphorylation sites were measured using primary antibodies against AMPK ^Thr172^ (#2535S, Cell Signaling), AMPKα2 protein (#G3013, Santa Cruz Biotechnology), p38 ^Thr180/Tyr182^ (#4511, Cell Signaling), p38 protein (#9212, Cell Signaling), CREB^Ser133^ (#9191, Cell Signaling), CAMKII (#3361, Cell Signalling), LC3A/B (#4108, Cell Signaling), p62 (#5114, Cell Signaling), ULK^Ser317^ (#12753, Cell Signaling), ULK^Ser757^ (#6888, Cell Signaling), ULK1 (#8054, Cell Signaling) and beta-actin (#4967, Cell Signaling). Equal loading was confirmed by similar β-actin content in the exercise training study and similar AMPKα2 and p38 protein content in the acute exercise protocol. The membranes were incubated in horse radish peroxidase conjugated secondary antibodies (Dako, Glostrup, Denmark) and protein and phosphorylation were visualized using LuminataTM Classico Western HRP Substrate (Millipore, Denmark). Band intensity was quantified using ImageQuant Las 4000 (GE Healthcare, Munich, Germany) and ImageQuant Imaging software. Protein content and phosphorylation were expressed in arbitrary units relative to control samples loaded on each site of each gel. Phosphorylation levels were normalized to the content of the target protein.

#### RNA isolation, reverse transcription and real-time PCR

Total RNA was isolated from crushed 15–20 mg muscle tissue by a modified guanidinium thiocyanate-phenol-chloroform extraction method (Chomczynski and Sacchi (1987); Pilegaard et al. 2000) except for the use of a TissueLyser (TissueLyser II, Qiagen, Valencia, CA, USA) for homogenization.

Superscript II RNase H^-^ system and Oligo dT (Invitrogen, Carlsbad, CA, USA) were used to reverse transcribe the mRNA to cDNA as described previously [8] Quantification of cDNA as a measure of mRNA content of a given gene was performed by real-time PCR using an ABI 7900 sequence-detection system (Applied Biosystems, Foster City, CA, USA). Primers and TaqMan probes were designed from mouse specific database (www.ensembl.org/) and Primer Express (Applied Biosystems) and are presented in Table 1. Self-designed TaqMan probes were labelled with 5´-6-carboxyfluorescein (FAM) and 3´-6-carboxy-N,N,N´,N´-tetramethylrhodamine (TAMRA) and obtained from TAG Copenhagen (Copenhagen, Denmark).

**Table 1:** Primer and probe sequences used for real-time PCR.

Gene	Forward primer	Reverse Primer	Amplicon size
Full Length PGC-1α	5' TCAAGCCAAACCAACAACTTTATCT 3'	5' GGTTCGCTCAATAGTCTTGTTCTCA 3'	97bp
NT-PGC-1α	5' TGCTTCGAAAAAGAAGTCCCATAC 3'	5' GGTCACTGGAAGATATGGCACAT 3'	132bp
Cyt C	5' TGCCCAGTGCCACACTGT 3'	5' CTGTCTTCCGCCCGAACA 3'	80bp
HKII	5' CTGTCTACAAGAAACATCCCCATTT 3'	5' CACCGCCGTCACCATAGC 3'	134bp
PGC-1α A	5' TGCATGAGTGTGTGCTGTGTGTC 3'	5' CACCAACCAGAGCAGCACACT 3'	138bp
PGC-1α B	5' GAGTATCTGCACTCCAGCAGAAT 3'	5' TCACCAACCAGAGCAGCACATT 3'	89bp
PGC-1α C	5' GTAACCGGAGGCATTCTCTCC 3'	5' CACCAACCAGAGCAGCACACA 3'	65bp
Gene	Probe		
Full Length PGC-1α	5' CACCAAATGACCCCAAGGGTTCCC 3'		
NT-PGC-1α	5' AAACAAATTTGGTGACTCTGGGGTC 3'		
Cyt C	5' AGGCAAGCATAAGACTGGACCAAATCTCCA 3'		
HKII	5' CAGTGAGGAGGCTGGTGCCCGA 3'		

Primers and TaqMan probe sequences used for real-time PCR. PGC-1α, peroxisome proliferator-activated receptor-ϒ coactivator-1α; NT, N-terminal truncated; Cyt c, cytochrome c; HKII, Hexokinase II.

**Fig 1:**
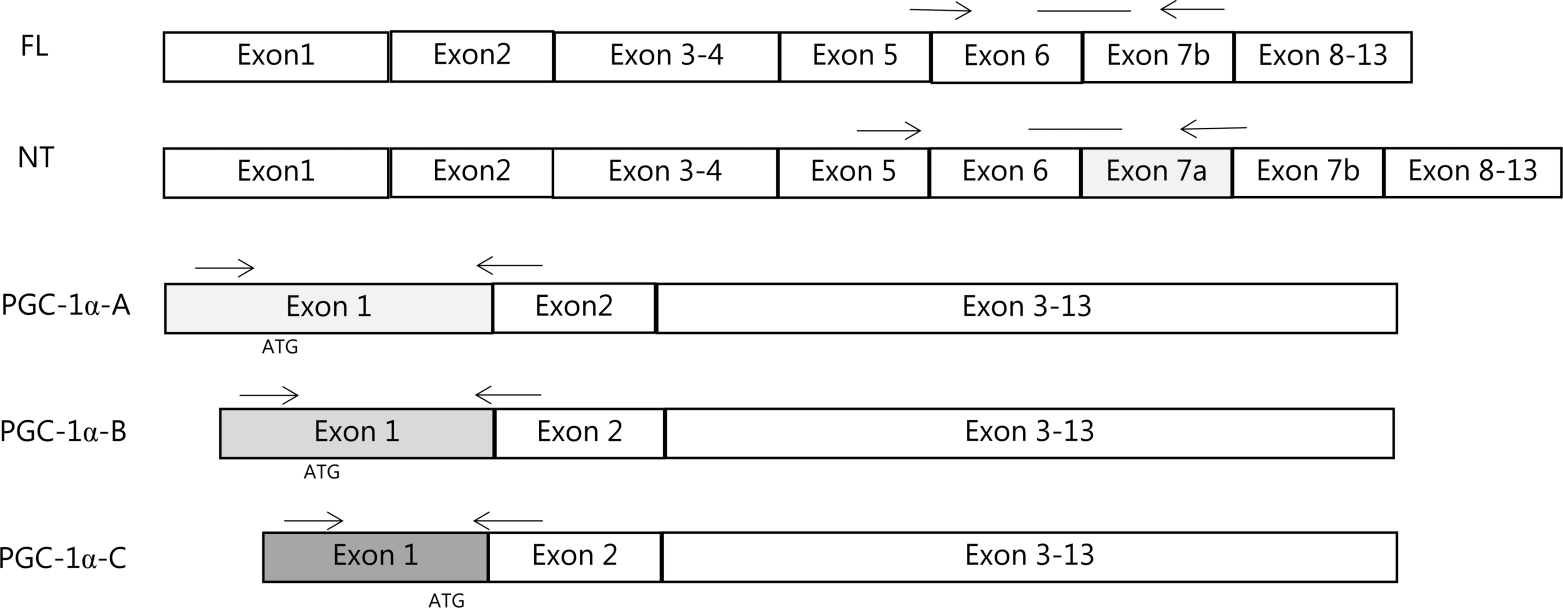
Fig 1 shows a schematic illustration of the positions of the primers and TaqMan probe sequences used for detection of the mRNA encoding the PGC-1α isoforms. FL primers and TaqMan probe detect the previously described PGC-1-α1/-a, PGC-1-α-b and PGC-1-α-c. The NT primers and Taqman probe detect the previously described NT-PGC-1-α-a, NT-PGC-1-α-b and PGC-1-α-c. The PGC-1α-A primers detect the previously described PGC-1-α1/-a and NT-PGC-1-α-a. The PGC-1α-B primers detect the previously described PGC-1-α-b, NT-PGC-1-α4/-b and PGC-1-α2. The PGC-1α-C primers detect the previously described PGC-1-α-c, NT-PGC-1-α-c and PGC-1-α3.

Real-time PCR was performed (Applied Biosystems, 7900 HT sequence detection system) in triplicates in a total reaction volume of 10 **μ**l using Universal Mastermix with UNG (Applied Biosystems). The obtained cycle threshold values reflecting the initial content of the specific transcript in the samples were converted to a relative amount by using standard curves constructed from a serial dilution of a pooled sample made from all samples. For each cDNA sample, the mRNA content of the given target was normalized to glyceraldehyde-3-phosphate desidrogenase (GAPDH) mRNA in the single exercise bout study and beta-actin mRNA in the exercise training study. PGC-1α isoform A, B and C were determined with SyberGreen (Applied Biosystems). The PGC-1α KO mice in the present study lacks exon 3,4,5 and therefore the PGC-1α isoforms NT, Full Length, A, B and C. Therefore PGC-1α mRNA of these isoforms are only measuered in WT mice. Fig 1 shows a schematic illustration of the positions of the primers and TaqMan probe sequences used for detection of the mRNA encoding the PGC-1α isoforms. FL primers and TaqMan probe detect the previously described PGC-1-α1/-a, PGC-1-α-b and PGC-1-α-c. The NT primers and Taqman probe detect the previously described NT-PGC-1-α-a, NT-PGC-1-α-b and PGC-1-α-c. The PGC-1α-A primers detect the previously described PGC-1-α1/-a and NT-PGC-1-α-a. The PGC-1α-B primers detect the previously described PGC-1-α-b, NT-PGC-1-α4/-b and PGC-1-α2. The PGC-1α-C primers detect the previously described PGC-1-α-c, NT-PGC-1-α-c and PGC-1-α3.

## Statistics

Values are presented as mean±SE. Two-way ANOVA was applied to evaluate the impact of genotype and exercise protocol in response to acute exercise as well as exercise training on plasma adrenaline, plasma lactate, muscle glycogen, mRNA, phosphorylation levels and protein content. A Student-Newman-Keul`s post hoc test was used to locate differences. A significance level of p<0.05 was chosen, and statistical calculations were performed using SigmaPlot Version 13.

## Results

### Single treadmill exercise bout

#### Plasma adrenaline and lactate

Plasma adrenaline was in WT mice higher (p<0.05) immediately after exercise than in REST in both LI an MI, with no difference between LI and MI, whereas no change was observed in PGC-1α KO. Plasma adrenaline was higher (p<0.05) in PGC-1α KO than WT in REST (Table 2).

**Table 2:** Single treadmill exercise bout.

	WT			KO		
	REST	LI	MI	REST	LI	MI
Plasma Adrenaline (nmol/L)	2.9 (±0.6)	7.7 (±1.6)*	7.2 (±1.0)*	8.9 (±1.9) #	8.9 (±1.8)	8.1 (±2.3)
Plasma Lactate (mmol/L)	3.3 (±0.2)	5.4 (±0.3)*	8.7 (±0.5)* ¤	3.2 (±0.2)	4.9 (±0.4) *	8.7 (±0.9) * ¤
Muscle Glycogen (mmol∙kg∙w^-1^)	25.4 (±2.1)	17.7 (±1.5)*	14.3 (±1.4)*	15.8 (±2.2) #	12.6 (±4.1) # *	6.4 (±1.4) # *

Plasma adrenaline concentration, plasma lactate concentration and quadriceps glycogen content in PGC-1α knockout (KO) and littermate wildtype (WT) control mice at rest (REST) or immediately after a single treadmill exercise bout at either low intensity (LI) or moderate intensity (MI). Values are mean ±SE, n = 8.

*Significantly different from REST within given genotype (p<0.05).

¤ Significantly different from LI within given genotype (p<0.05).

# Significantly different from WT within given group (p<0.05).

Plasma lactate was higher (p<0.05) immediately after the exercise than in REST in both WT and PGC-1α KO mice and there was no difference in plasma lactate between genotypes (Table 2).

#### Muscle glycogen

Muscle glycogen was lower (p<0.05) immediately after the exercise than in REST and was higher (p<0.05) in MI than LI in both WT and PGC-1α KO mice, with no difference in muscle glycogen between LI and MI. Muscle glycogen was lower (p<0.05) in PGC-1α KO mice than WT in all groups (Table 2).

#### Muscle protein content and phosphorylation

**Fig 2:**
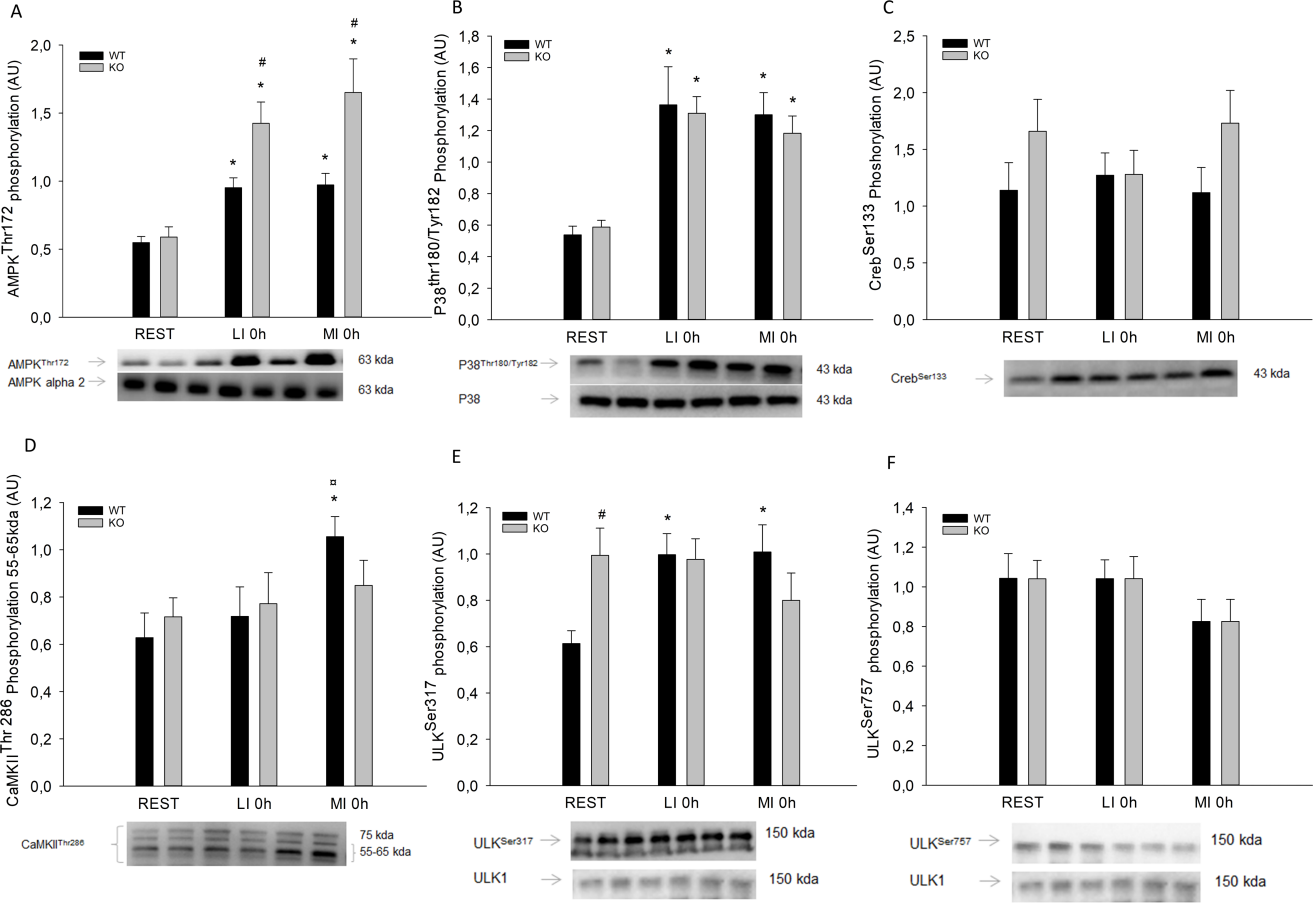
A) AMPK^Thr172^, B) P38^Thr180/Tyr182^, C) Creb^Ser133^, D) CaMKII^Thr286^ E) ULK^ser317^ and F) ULK^ser757^ phosphorylation in quadriceps from PGC-1α knockout (KO) and littermate wildtype (WT) control mice at rest (REST) or immediately after a single treadmill exercise bout at either low intensity (LI) or moderate intensity (MI). Values are mean ±SE, n = 8. *Significantly different from REST within given genotype (p<0.05). ¤ Significantly different from LI within given genotype (p<0.05). # Significantly different from WT within given group (p<0.05).

Muscle AMPK^Thr172^ phosphorylation was higher (p<0.05) immediately after the exercise than in REST in both WT (1.5 fold) and PGC-1α KO mice (2.5–3 fold) with no differences between LI and MI. Muscle AMPK^Thr172^ phosphorylation was higher (p<0.05) in PGC-1α KO mice than in WT in both LI and MI (Fig 2A). There were no differences in AMPKα2 protein content between groups or genotypes.

Muscle p38 mitogen-activated protein kinase (p38)^Thr180/Tyr182^ phosphorylation was 2-fold higher (p<0.05) immediately after the exercise bout than REST in both WT and PGC-1α KO mice, with no differences between LI and MI. There were no differences in p38^Thr180/Tyr182^ phosphorylation between the genotypes in any of the groups (Fig 2B), and no differences in p38 protein content between groups or genotypes.

There were no differences in muscle CaMK/cAMP-response element binding (Creb)^Ser133^ phosphorylation in any of the groups or between genotypes (Fig 2C).

Muscle Ca^2+^ /calmodulin-dependent protein kinase II (CaMKII)Thr 286 band 55–65 kda phosphorylation in WT was 1.5-fold higher (p<0.05) immediately after MI than in REST and LI, while there was no difference between groups in PGC-1α KO mice (Fig 2D). There were no differences in muscle CaMKII^Thr 286^ band 75 kda phosphorylation between groups or genotypes.

To obtain additional information regarding autophagy, the protein content and phosphorylations of the autophagy marker ULK1 were determined. There was no effect of acute exercise or genotype on the inhibitatory mTOR ULK phosphorylation site (ULK^Ser757^) in either exercise protocol (Fig 2E), while the activating AMPK phosphorylation site (ULK^Ser317^) was 1.4 fold higher (P<0.05) immediately after exercise than in REST in both LI and MI within WT mice, but not PGC-1α KO mice. In addition, ULK^Ser317^ phosphorylation was 1.4 fold higher (P<0.05) in PGC-1α KO mice than WT at REST (Fig 2F). There were no differences in ULK1 protein content between groups or genotypes.

**Fig 3:**
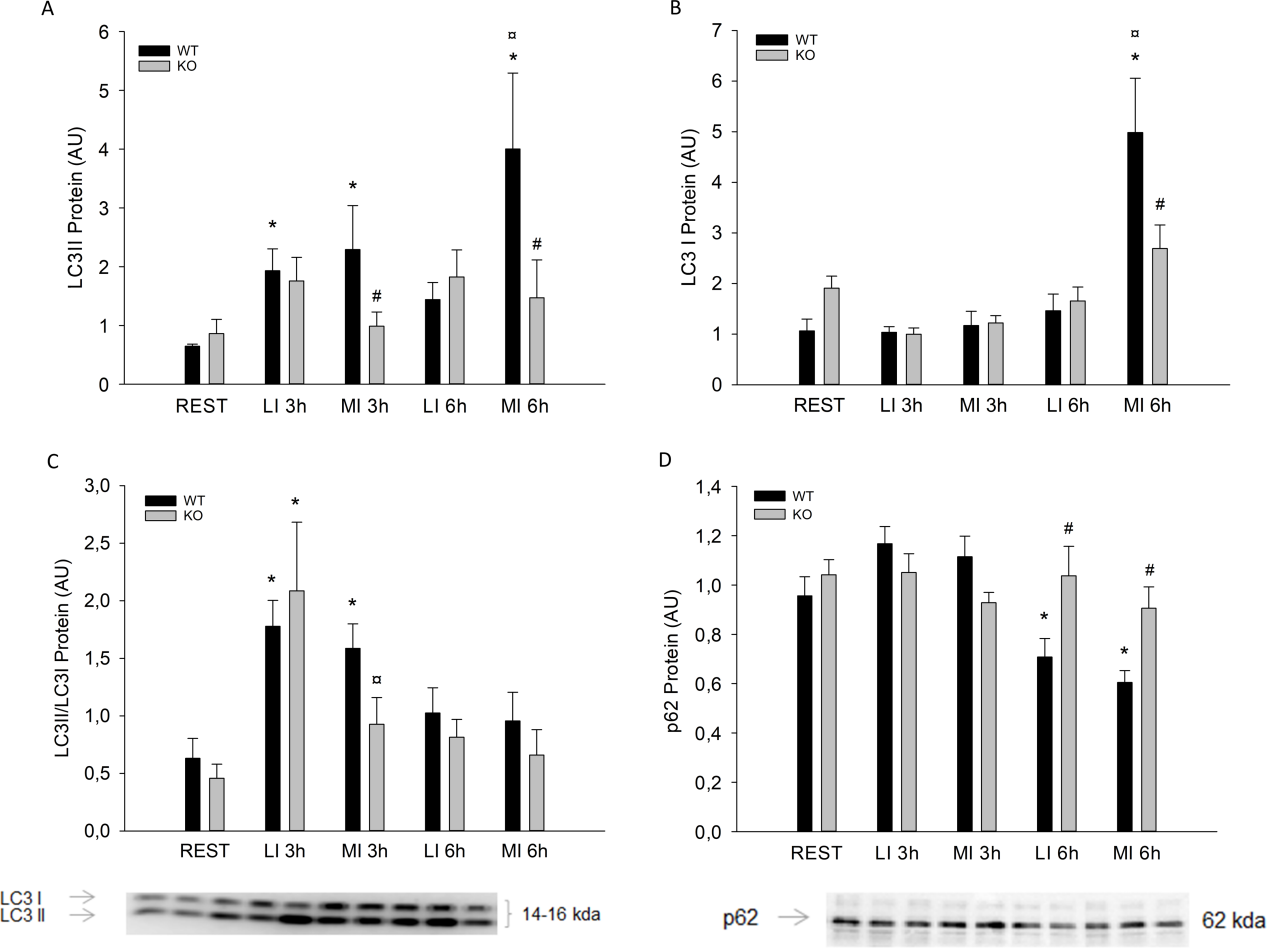
A) LC3II B) LC3I protein content, C) LC3II/LC3I Ratio and D) P62 protein content from PGC-1α knockout (KO) and littermate wildtype (WT) control mice at rest (REST), 3h and 6h after a single treadmill exercise bout at either low intensity (LI) or moderate intensity (MI). Values are mean ±SE, n = 8. *Significantly different from REST within given genotype (p<0.05). ¤ Significantly different from LI within given genotype (p<0.05). # Significantly different from WT within given group (p<0.05).

Muscle LC3II protein content was 2-3-fold higher (p<0.05) 3h after the exercise bout than in REST in WT mice only, with no differences between LI and MI. In addition, muscle LC3II protein content was 5-fold higher (p<0.05) 6h after MI than in resting controls in WT mice only. Muscle LC3II protein content was 50% lower (p<0.05) in PGC-1α KO mice than in WT in MI (Fig 3A). Muscle LC3I protein content was 4 fold higher (p<0.05) 6h after the MI exercise bout than in REST in WT mice only (Fig 3B). The LC3II/LC3I ratio was in WT 3 fold higher (p<0.05) 3h after exercise in both LI and MI and in PGC-1α KO mice 4 fold higher (p<0.05) 3h after exercise than in REST only in LI. There were no differences at 6h in any of the groups (Fig 3C).

Muscle p62 protein content was 30–35% lower (P<0.05) at 6h after both LI and MI than REST in WT mice, while there was no effect of acute exercise on p62 protein in PGC-1α KO mice. In addition, p62 protein content was approximately 35% higher (P<0.05) in PGC-1α KO mice than WT mice 6h after both LI and MI (Fig 3D).

#### Muscle mRNA levels

**Fig 4:**
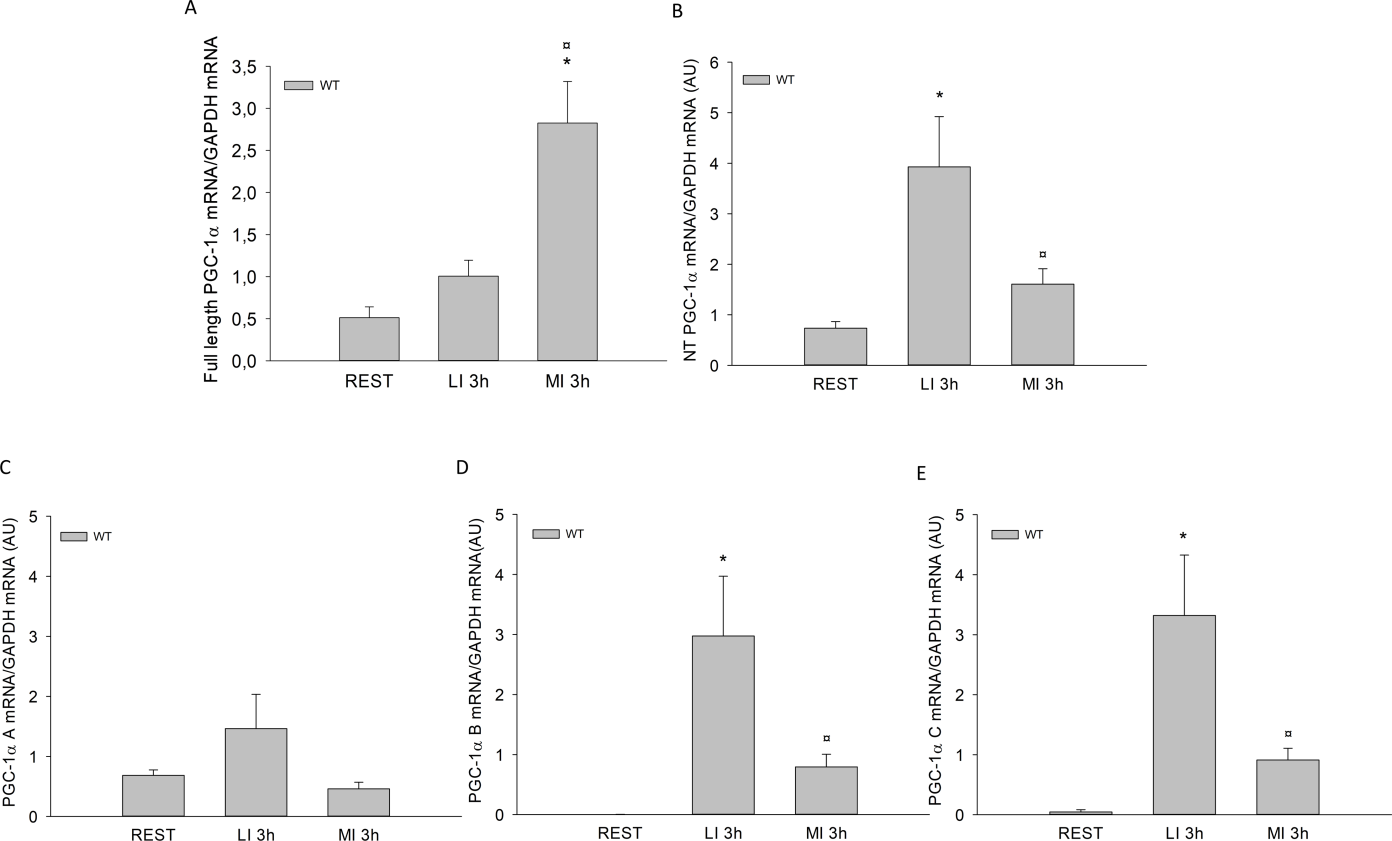
A) Full Length PGC-1α, B) NT PGC-1α, C) PGC-1α A, D) PGC-1α B and E) PGC-1α C mRNA content in quadriceps from WT control mice at rest (REST) or 3h after a single treadmill exercise bout at either low intensity (LI) or moderate intensity (MI). Values are mean ±SE with n = 8. *Significantly different from REST within given genotype (p<0.05). ¤ Significantly different from LI within given genotype (p<0.05).

Muscle full length PGC-1α mRNA content was 4- and 3-fold higher (p<0.05) 3h after MI than in REST and than 3h after LI, but not different between LI and REST (Fig 4A).

Muscle NT PGC-1α mRNA content was 4 fold and 2 fold higher (p<0.05) 3h after LI than in REST and than 3h after MI, respectively, but not different between MI and REST (Fig 4B).

There were no differences in muscle PGC-1α A mRNA content between groups (Fig 4C). Muscle PGC-1α B mRNA content was 600 fold and 3 fold higher (p<0.05) 3h after LI than REST and than 3h after MI, respectively, but not different between MI and REST (Fig 4D). Muscle PGC-1α C mRNA content was 60 fold and 3 fold higher (p<0.05) 3h after LI than REST and than MI, respectively, but not different between MI and REST (Fig 4D).

**Fig 5:**
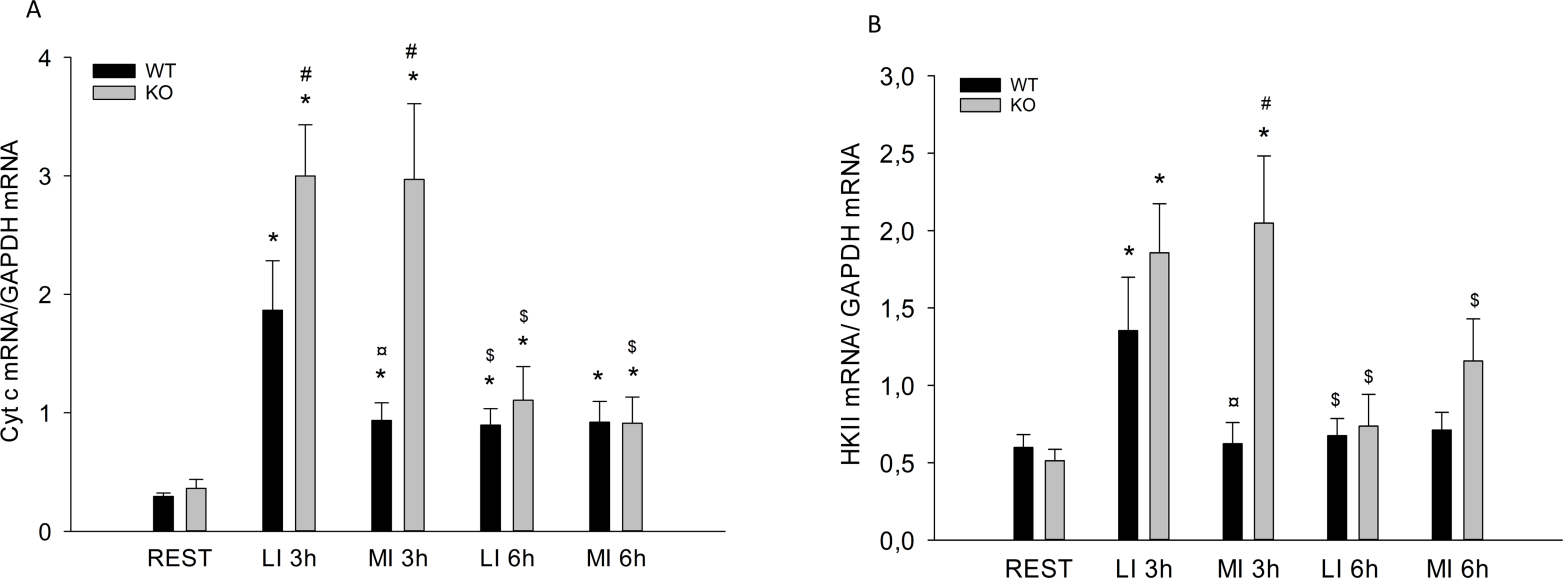
A) Cytochrome C (Cyt C) and B) HexokinaseII (HKII) mRNA content in quadriceps from PGC-1α knockout KO and littermate wildtype (WT) control mice at rest (REST), 3h and 6h after a single treadmill exercise bout at either low intensity (LI) or moderate intensity (MI). Values are mean ±SE, n = 8. *Significantly different from REST within given genotype (p<0.05). ¤ Significantly different from LI within given genotype (p<0.05). $ Significantly different from 3h within given group and genotype (p<0.05). # Significantly different from WT within given group (p<0.05).

Cyt C mRNA content was 2–7 fold higher (p<0.05) 3h after MI and LI than REST in WT and PGC-1α KO mice. Cyt C mRNA was 50% lower (p<0.05) 3h after MI than 3h after LI in WT mice. Muscle Cyt C mRNA was 2 fold higher (p<0.05) 6h after exercise than in REST in both WT and PGC-1α KO mice (Fig 5A).

Muscle Hexokinase (HK) II mRNA content was 3 fold higher (p<0.05) 3h after LI in WT and 3h after MI and LI in PGC-1α KO mice than in REST. The HKII mRNA content was 60% lower (p<0.05) 3h after MI than 3h after LI in WT mice. There were no differences in muscle HKII mRNA content between groups or genotypes 6h after exercise (Fig 5B).

### Exercise training

#### Body weight and composition

**Fig 6:**
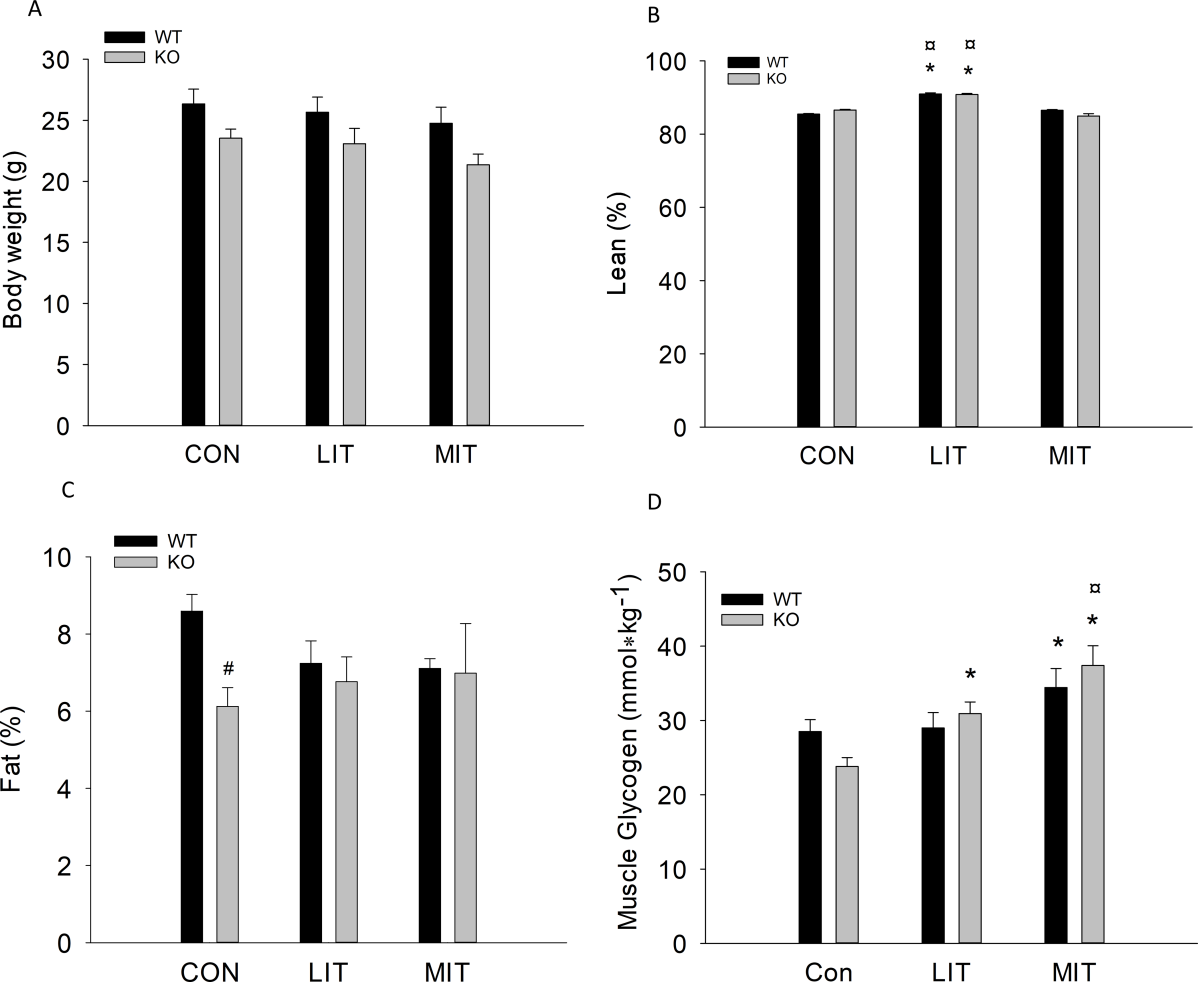
A) Body weight, B) lean percentage (%), C) fat percentage (%) and D) quadriceps glycogen content in PGC-1α knockout (KO) and littermate wildtype (WT) control mice after 5 weeks untrained (CON) or exercise training at either low intensity (LIT) or moderate intensity (MIT). Values are mean ±SE, n = 10. *Significantly different from CON within given genotype (p<0.05). ¤ Significantly different from LIT within given genotype (p<0.05). # Significantly different from WT within given group (p<0.05).

There was no difference in body weight between groups or genotypes (Fig 6A). Lean body mass was higher (p<0.05) in LIT than in CON and higher in LIT than in MIT in both WT and PGC-1α KO mice, while there was no difference between MIT and CON. Moreover, there was no difference in lean body mass between genotypes (Fig 6B). There was no difference in fat mass percentage between groups either in WT or PGC-1α KO mice. However, the fat mass percentage in CON was lower (p<0.05) in PGC-1α KO mice than in WT mice (Fig 6C).

#### Muscle glycogen

In WT, muscle glycogen was higher (p<0.05) in MIT than CON. In PGC-1α KO, muscle glycogen was higher (p<0.05) in both LIT and MIT than in CON and also higher (p<0.05) in MIT than LIT (Fig 6D).

#### Endurance running

**Fig 7:**
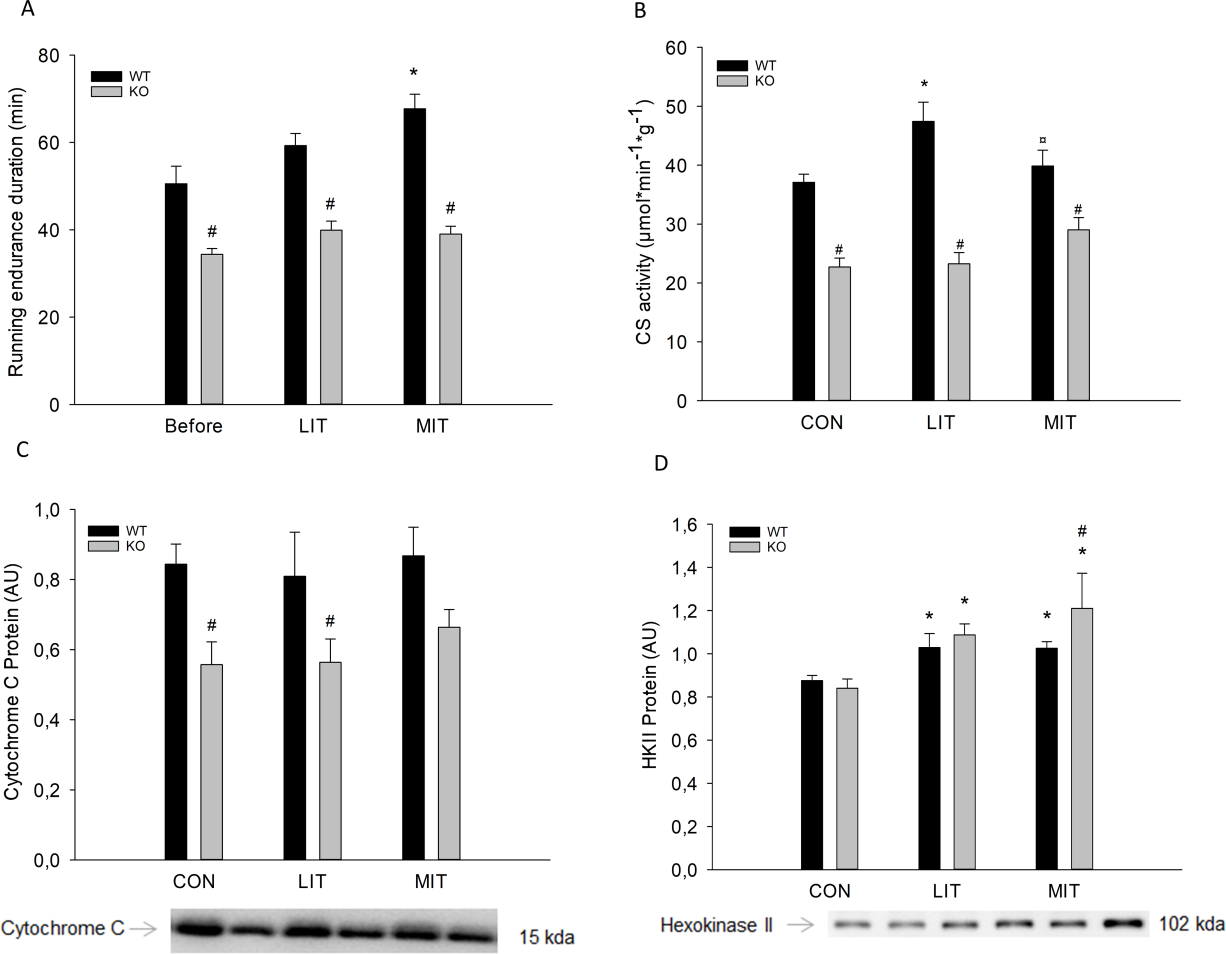
A) Running endurance duration (minutes) during a running test performed in week 1 and 5 of the intervention, and muscle B) Citrate synthase activity (CS), C) Cytochrome C protein, and D) Hexokinase II (HKII) protein content from PGC-1α knockout (KO) and littermate wildtype (WT) control mice after 5 weeks untrained (CON) or exercise training at either low intensity (LIT) or moderate intensity (MIT). Values are mean ±SE, n = 10. *Significantly different from CON within given genotype (p<0.05). ¤ Significantly different from LIT within given genotype (p<0.05). # Significantly different from WT within given group (p<0.05).

In MIT, time to exhaustion during the running test was improved (p<0.05) by exercise training in WT, but not in PGC-1α KO mice. Time to exhaustion was shorter (p<0.05) in PGC-1α KO mice than in WT mice in all groups (Fig 7A).

#### CS activity

CS activity was 1.2 fold higher (p<0.05) in LIT than CON within WT, while there was no difference in CS activity within PGC-1α KO mice. Furthermore, CS activity was 20–40% lower (p<0.05) in PGC-1α KO mice than in WT in all groups (Fig 7B).

#### Muscle protein content

There was no difference in Cyt C protein content between groups either in WT or in PGC-1α KO mice. Cyt C protein content was 30% lower (p<0.05) in PGC-1α KO mice than in WT mice in CON and LIT (Fig 7C). HKII protein content was 1,2–1,3 fold higher (p<0.05) after five weeks of LIT and MIT exercise training than in CON in both WT and PGC-1α KO mice. HKII protein content was 1,2 fold higher (p<0.05) in PGC-1α KO than WT in MIT (Fig 7D).

**Fig 8:**
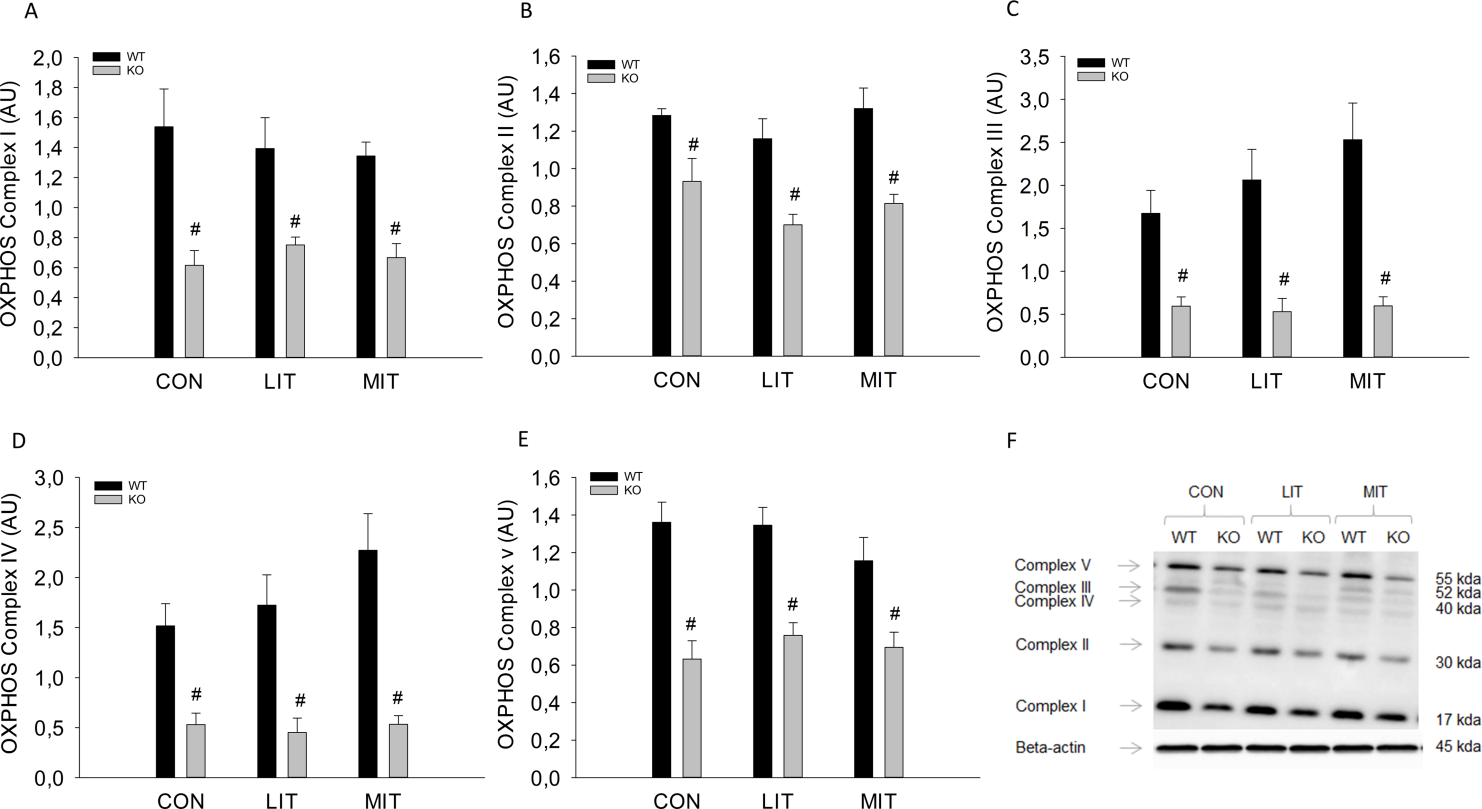
A) OXPHOS Complex I protein content (AU), B) OXPHOS Complex II protein content (AU), C) OXPHOS Complex III protein content (AU), D) OXPHOS Complex IV protein content (AU) E) OXPHOS Complex V protein content (AU) and F) representative Blots from PGC-1α knockout (KO) and littermate wildtype (WT) control mice after 5 weeks untrained (CON) or exercise training at either low intensity (LIT) or moderate intensity (MIT). Values are mean ±SE, n = 10. # Significantly different from WT within given group (p<0.05).

There was no difference in OXPHOS Complex I, II, III, IV and V protein content between groups either in WT or in PGC-1α KO mice. OXPHOS Complex I, II, III, IV and V protein content was 15–70% lower (p<0.05) in PGC-1α KO mice than in WT mice in all groups (Fig 8A–8F).

**Fig 9:**
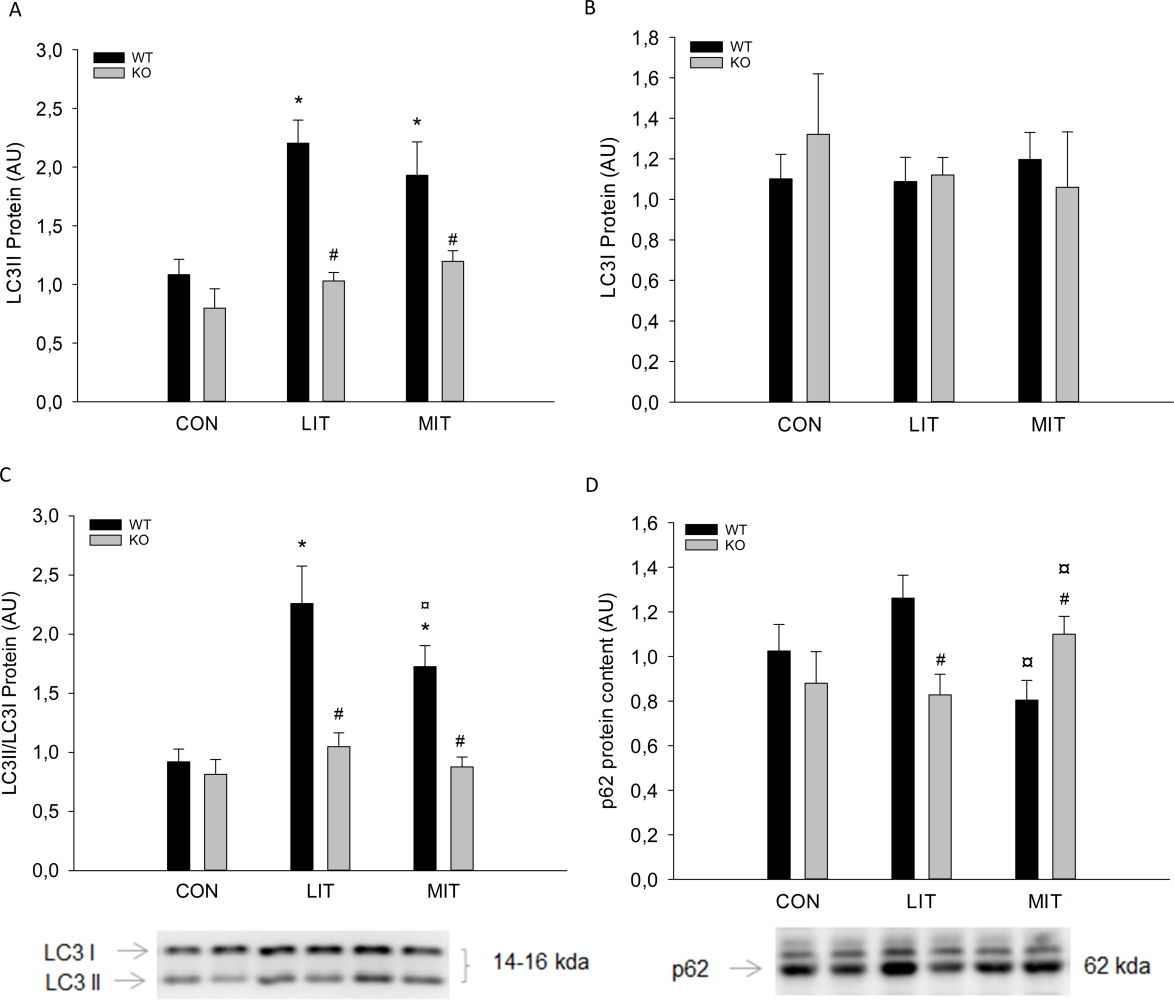
A) LC3II B) LC3I protein content, C) LC3II/LC3I Ratio D) and p62 protein content (AU) in quadriceps from PGC-1α knockout KO and littermate wildtype (WT) control mice after 5 weeks untrained (CON) or exercise training at either low intensity (LIT) or moderate intensity (MIT). Values are mean ±SE, n = 10. *Significantly different from CON within given genotype (p<0.05). ¤ Significantly different from LIT within given genotype (p<0.05). # Significantly different from WT within given group (p<0.05).

LC3II protein content was 1.8–2 fold higher (p<0.05) after LIT and MIT than in CON only in WT mice. LC3II protein content was 40–50% lower (p<0.05) in PGC-1α KO mice than in WT mice in LIT and MIT (Fig 9A). There were no differences in muscle LC3I protein content between any of the groups or between genotypes (Fig 9B). The LC3II/LC3I ratio was 2–2,3 fold higher (p<0.05) after five weeks of LIT and MIT exercise training than CON only in WT mice. The LC3II/LC3I ratio was 50% lower (p<0.05) in PGC-1α KO mice than in WT mice in LIT and MIT (Fig 9C).

#### Muscle mRNA levels

There was in WT mice no difference in Full Length, NT, A, B or C PGC-1α mRNA content between groups after LIT or MIT exercise training (Table 3).

**Table 3:** Exercise training–PGC-1α mRNA isoforms.

	WT		
	CON	LIT	MIT
PGC-1α Full Length (AU)	1.29 (±0.16)	1.46 (±0.21)	1.75 (±0.17)
PGC-1α NT (AU)	1.27 (±0.09)	1.61 (±0.21)	1.47 (±0.15)
PGC-1α A (AU)	0.99 (±0.05)	1.20 (±0.04)	1.12 (±0.06)
PGC-1α B (AU)	0.19 (±0.05)	0.17 (±0.04)	0.21 (±0.06)
PGC-1α C (AU)	0.27 (±0.09)	0.35 (±0.11)	0.61 (±0.16)

Full Length PGC-1α, NT PGC-1α, PGC-1α A, PGC-1α B and PGC-1α C mRNA content in quadriceps from wildtype (WT) control mice after 5 weeks untrained (CON) or exercise training at either low intensity (LIT) or moderate intensity (MIT). Values are mean ±SE, n = 10.

## Discussion

The main findings of the present study are that a single exercise bout increased LC3II protein in skeletal muscle in an intensity and PGC-1α dependent manner, and a period of exercise training increased LC3II protein only in WT mice independent of exercise intensity. Furthermore, acute exercise increased mRNA’s encoding metabolic markers in skeletal muscle independent of PGC-1α and with most marked changes in response to low intensity exercise. These responses were only in part reflected in exercise training metabolic adaptations. In addition, exercise-induced PGC-1α isoform mRNA responses were intensity and/or volume dependent supporting the possibility that specific isoforms exert exercise protocol specific acute mRNA responses.

The present finding that acute exercise increased LC3II protein and LC3II/LC3I ratio in skeletal muscle of WT mice and that the response in LC3II protein in WT was intensity dependent are in line with previous findings [21,23,51–54]. However, the more marked increase in LC3II protein after moderate intensity exercise than low intensity exercise at 6h of recovery is novel, because exercise has previously been reported to decrease the LC3II/LC3I ratio in human skeletal muscle [54]. Whether these opposite responses are due to species differences remains to be determined. In addition, the observed increase in ULK^Ser317^ phosphorylation immediately after exercise and the decrease in p62 protein in recovery from exercise in both protocols are in accordance with the increased LC3II protein after exercise together indicating exercise-induced enhanced autophagy. The finding that LC3II already increased 3h after exercise, while the p62 protein decrease was evident at 6h after exercise shows for the first time that these commonly used autophagy markers can have different timing of the exercise-induced responses. Moreover, despite the similar response in p62 protein in LI and MI 6h after exercise it is still possible that p62 protein may differ between protocols at a later time point and hence exhibit an intensity dependent regulation as observed for LC3II in the present study, although this remains to be determined. Of notice is that the increase in LC3II protein in the present study was paralleled by a similar intensity dependent increase in LC3I protein content, and therefore no change in the LC3II/LC3I ratio was observed. This provides evidence of a fast upregulation of LC3I protein with concomitant increased capacity for LC3I lipidation and hence autophagy in response to moderate intensity but not low intensity exercise. On the other hand, the increase in LC3II protein and LC3II/LC3I ratio at 3h of recovery was independent of intensity/volume suggesting that this response is only lipidation of already existing LC3I. The current findings that LC3I, LC3II and LC3II/LC3I each demonstrated exercise protocol dependent time course responses to acute exercise and that p62 decreased at 6h, but not 3h after exercise underline the strength of applying multiple sampling time points. In addition, while several studies have shown exercise-induced regulation of LC3II and LC3II/LC3I in human and mouse skeletal muscle immediately after exercise or 1h after exercise [23,51,52,54], the present observations that both LC3I and LC3II protein increased markedly and p62 protein decreased late in recovery, as also in part previously shown[21], indicate a long-lasting autophagy response after exercise.

The present observation that exercise training increased LC3II protein in WT is in accordance with a previous mouse study [55], which together potentially reflects elevated basal autophagy flux in skeletal muscle after exercise training. However, the lack of change in p62 protein and ULK phosphorylation with both LIT and MIT does as such not support an exercise training-induced increase in autophagy flux. On the other hand, although exercise training has been reported to decrease p62 protein in mouse skeletal muscle [55], unchanged or even increased p62 protein has also been reported concomittant with increased LC3II or LC3II/LC3I [21,23,55]. These differences may in part be related to the investigated muscle types and emphasize the challenge in interepreting exercise training-induced adaptations in autophagy markers. Of notice is that the increased LC3II protein involves a post translational modification rather than new synthesis of a given protein [56] and was not associated with increased LC3I protein with exercise training as previously reported in mice [55]. Furthermore, the lack of an increase in LC3I protein with exercise training, although LC3I increased 6h after a single bout of moderate intensity exercise, is not in line with the suggested mechanism behind long-term protein adaptations [2,57]. However, the increase in LC3II protein without change in LC3I with moderate exercise training may suggest that exercise training did increase the production of LC3I, but that this then was lipidated to LC3II. On the other hand, regulation of the factors mediating the lipidation of LC3I with exercise training also seems likely, as only the acute moderate intensity exercise increased LC3I late in recovery with potential cumulative effects. Previous studies have both used LC3II protein content and the LC3II/LC3I ratio as an indication of autophagy flux [21,55]. The present findings that the LC3II/LC3I ratio increased less with MI than LI, while exercise training increased LC3II protein independent of intensity/volume underline that the impact of exercise training intensity on basal autophagy flux remains to be determined.

The present findings, that exercise training increased LC3II protein and LC3II/LC3I without adaptations in the content of oxidative proteins, are not in accordance, with the observation in mice that elevated basal autophagy in a mixed muscle with voluntary wheel running exercise training, only occurred if a parallel increase in oxidative proteins took place [55]. This may suggest, that the present changes in LC3II and LC3II/LC3I and the potential elevated basal autophagy with exercise training without associated changes in the content of OXPHOS and Cyt c protein, are related to the use of treadmill exercise twice a day. Hence lack of sufficient recovery may have elicited the dissociation between autophagy and metabolic adaptations, although additional experiments are required to draw final conclusions on this matter. In addition, the observation that running endurance increased after moderate intensity exercise training without an associated increase in OXPHOS protein or CS activity may suggest that other factors than skeletal muscle oxidative capacity determined exercise performance in the current protocol. This may suggest that the improved running endurance was related to the observed effects on autophagy and hence potentially mitochondrial quality control“.

The observations that plasma adrenaline increased similarly, muscle glycogen levels decreased and both AMPK^Thr172^, p38^Thr180/Tyr182^ and ULK^Ser317^ phosphorylation increased to the same level in both protocols in WT mice, suggests that the differences in LC3I and LC3II protein as well as Cyt c and HKII mRNA between protocols were not related to these parameters. This is not in accordance with the previous suggestion that AMPK mediated the exercise-induced autophagy response in humans[54], while another study reports no effect of AMPK [58]. However, the faster use of muscle glycogen in MI than LI and the higher level of plasma lactate in MI than LI indicate higher glycolytic flux. And, hence, differences in the intracellular intermediates that may have regulatory roles. Furthermore, the increased CaMKII^Thr285^ phosphorylation after exercise only in MI likely reflecting enhanced calcium levels may support an impact of calcium-CaMK signaling in the observed protocol specific responses.

The present observation that the exercise-induced LC3II protein response was blunted in PGC-1α KO mice is in agreement with the previous finding that PGC-1α was required for an exercise induced increase in LC3II protein after exhaustive exercise [23] and after prolonged low intensity exercise [21]. However, the present observations that PGC-1α was mandatory for the increase in LC3II protein and decrease in p62 protein in response to both low and moderate non-exhaustive acute exercise, that ULK^Ser317^ phosphorylation increased only in WT mice have not previously been reported. Furthermore the finding that PGC-1α was required for the increase in LC3II protein and LC3II/LC3I with exercise training and that lack of PGC-1α influenced the p62 protein level after exercise training are novel. Moreover, the findings that PGC-1α was required for the increase in CS activity with LIT and exercise endurance with MIT are in agreement with previous studies [6,14,20]. Together this supports that PGC-1α mediated coordination of exercise training adaptations in skeletal muscle with increased mitochondrial content and autophagy, as previously suggested [22]. Furthermore, the increase in HKII protein content in PGC-1α KO mice both with LIT and MIT underlines that the PGC-1α KO mice in the present study did exhibit metabolic adaptations.

The present observations, that acute exercise increased Cyt c and HKII mRNA in PGC-1α KO mice in both exercise protocols, show that these responses were independent of PGC-1α, which is opposite of a previous study [14], but in line with others [14,59]. Moreover, the findings in the current study, that both the low intensity and the moderate intensity exercise protocol elicited the mRNA responses when PGC-1α was lacking, suggests that the differences between studies are not entirely related to the differences in intensity and duration of the applied exercise protocols. The use of different sampling time points provides a potential explanation of the different findings. In addition, it should be noted that the PGC-1α KO mice exercised at a higher relative exercise intensity than the WT and that this is a likely explanation for obtaining a higher level of cyt c mRNA in the PGC-1α KO than the WT in recovery from exercise. Together this supports that PGC-1α is not mandatory for exercise-induced cyt c mRNA regulation in mouse skeletal muscle when using the current exercise protocols.

The present observation, that the phosphorylation of the intracellular energy sensor AMPK was higher in PGC-1α KO mice than WT immediately after both LI and MI exercise, indicates that the PGC-1α KO mice were more metabolically challenged than WT mice, and that the PGC-1α KO mice exercised at a higher relative intensity. Hence, the observed PGC-1α dependent regulation of autophagy markers is despite that the mice exercised at a higher relative intensity underlining the impact of PGC-1α in this response. In addition, the current PGC-1α independent Cyt c mRNA response, opposite of previous findings may be related to the higher relative exercise intensity in PGC-1α KO mice than WT mice. Therefore, it cannot be ruled out that PGC-1α plays a role in acute regulation of this mRNA when the exercise is performed at the same relative intensity. The present observations that the resting plasma adrenaline concentration was elevated in PGC-1α KO mice relative to WT and did only change in response to exercise in WT are novel and may have contributed to the observed genotype differences, although this remains to be clarified.

The present findings, that acute exercise elicited isoform specific mRNA responses of the three isoforms deviating at the N terminal with pronounced increases in isoform B and C and no change in isoform A as well as increase in NT-PGC-1α mRNA, are in accordance with a previous study [49]. However, the observations that only LI induced isoform B, C and NT-PGC-1α in the present study are different from the previous study reporting the highest induction of these isoforms at the highest running speed [48]. These differences may be explained by the use of different muscles, different mouse strains, sampling time points and volume of the exercise. However, the responses in the present and previous study [48,49] also underline the overall robustness of an exercise-induced PGC-1α isoform specific induction in skeletal muscle. In addition, the observed marked induction of full length PGC-1α isoform only with the moderate intensity protocol has to our knowledge not been reported previously and suggests that this isoform is regulated differently by exercise intensity than the other four isoforms. Of notice is that the observed mRNA responses of PGC-1α isoform B, C and NT with induction only in response to LI are in accordance with the observed exercise training induced adaptation in CS activity only after LIT. On the other hand, the induction of full length PGC-1α mRNA only in response to MI is in line with the previously reported intensity dependency of the PGC-α mRNA response in humans [27,29] as well as the increase in CaMKII phosphorylation and the observed acute regulation of LC3I and LC3II protein late in recovery in the present study.

In conclusion, acute exercise elicited an intensity dependent increase in LC3I and LC3II protein, but an intensity independent decrease in p62 protein in skeletal muscle late in recovery and increased LC3II with exercise training independent of differences in exercise intensity and volume. Furthermore, PGC-1α was required for the acute exercise-induced regulation of LC3I, LC3II and p62 protein, for the exercise training-induced regulation of LC3I and LC3II protein and lack of PGC-1α influenced the p62 protein content after exercise training. In addition, exercise-induced mRNA responses of PGC-1α isoforms were intensity dependent suggesting that specific PGC-1α isoforms mediates exercise intensity dependent adaptations. Taken together these findings indicate that exercise intensity affected autophagy markers differently in skeletal muscle and suggest that PGC-1α regulates exercise training-induced autophagy in skeletal muscle potentially in a PGC-1α isoform specific manner.

## Abbreviations

AMPK: AMP-activated protein kinase
ATF6: Activating transcription factor 6
CaMKII: Ca^2+^ /calmodulin-dependent protein kinase II
CON: Control
COX: Cytochrome C Oxidase
Creb: cAMP response element binding protein
CS: Citrate Synthase
Cyt C: Cytochrome C
GAPDH: Glyceraldehyde 3-phosphate dehydrogenase
HKII: Hexokinase II
KO: knockout
LC3: Microtubule-associated protein 1A/1B-light chain 3
LI: Low Intensity exercise
LIT: Low Intensity Training
MI: Moderate Intensity Exercise
MIT: Moderate Intensity Training
MKO: Muscle specific knockout
NT: N-terminal
OXPHOS: Oxidative phosphorylation
p38: p38 mitogen activated protein kinase
PGC-1α: Peroxisome proliferator-activated receptor-gamma coactivator 1 alpha
REST: resting control group
ROS: Reactive Oxygen Species
SDS: Sodium dodecyl sulfate-polyacrylamide
WT: Wildtype
